# The Value of Fecal Microbiota Transplantation in the Treatment of Ulcerative Colitis Patients: A Systematic Review and Meta-Analysis

**DOI:** 10.1155/2018/5480961

**Published:** 2018-04-03

**Authors:** Yantian Cao, Bangjie Zhang, Yuanyuan Wu, Qingzhi Wang, Jie Wang, Fangfang Shen

**Affiliations:** ^1^Department of Gastroenterology, The Third Affiliated Hospital of Xinxiang Medical University, Hua Lan Avenue, Xinxiang, Henan Province 453003, China; ^2^Department of Oncology, The Third Affiliated Hospital of Xinxiang Medical University, Hua Lan Avenue, Xinxiang, Henan Province 453003, China; ^3^School of Automation, Key Laboratory of Image Processing and Intelligent Control of Education Ministry of China, Huazhong University of Science and Technology, Wuhan, Hubei Province 430022, China; ^4^The Key Laboratory for Tumor Translational Medicine, The Third Affiliated Hospital of Xinxiang Medical University, Hua Lan Avenue, Xinxiang, Henan Province 453003, China

## Abstract

**Background and Aims:**

Fecal microbiota transplantation (FMT) has challenged the traditional management of ulcerative colitis (UC) in recent years, while it remained controversial. We aimed to provide a systematic protocol of FMT treatment on UC.

**Methods:**

Studies reporting on FMT treatment in UC patients were performed. A fixed-effect model was used to assess the efficacy of FMT.

**Results:**

Eighteen studies were enrolled (*n* = 446). A pooled proportion of patients who received FMT had a significant efficacy compared to the placebo group (odds ratio (OR): 2.73, *P* = 0.002) with a low risk of heterogeneity (*P* = 0.59, *I*^2^ = 0%). The Mayo score decreased to 5 points in a state of mild–moderate activity after FMT treatment, and the optimal range of the Mayo score baseline was 6–9 for FMT administration. Then, the baseline of the Shannon diversity index (SDI) had a negative correlation with the clinical response rate (*R* = −0.992, *P* = 0.08) or remission rate (*R* = −0.998, *P* = 0.036), and the optimal diversity of bacteria was at 7 days to one month. Moreover, the colonoscopy delivery and unrelated fecal donor had slight superiorities of FMT treatment.

**Conclusion:**

FMT treatment had a higher efficacy and shorter time-point of early assessment of effectiveness on UC patients compared to traditional therapies. And the optimal FMT delivery and donor were colonoscopy delivery and unrelated donor in clinical practice.

## 1. Introduction

Ulcerative colitis (UC), a subtype of inflammatory bowel disease (IBD), is a chronic, relapsing, and remitting disease characterized by the aggressive inflammation contributing to the destruction of the colonic mucosa [1]. Its main symptoms include bloody diarrhea, abdominal pain, urgent, and tenesmus [2, 3], which produce a miserable influence on the quality of life. Meanwhile, about 3 million of people suffered from it [4], while the etiologies of UC still have remained unclear and were involved in immunologic, genetic, environmental, and gut microbial changes. Several studies demonstrated that the abnormal changes in gut microbiota (e.g., Firmicutes and Bacteroidetes) reduced the ability of the intestinal environment to fight pathogens and can be relevant with some disease conditions [5, 6].

Fecal microbiota transplantation (FMT) was first reported formally in 1958 with treatment of pseudomembranous colitis [7]. Afterward, FMT has been applied for refractory *Clostridium difficile* infection (CDI) on the basis of the rebuilding of abnormal bacterial species of the human gut [8], which is more effective than antibiotics for recurrent CDI patients (87~90%) [9, 10]. IBD patients were at a higher risk for microbiota disorder in the intestinal tract; therefore, FMT has been regarded as a potential treatment for IBD management and showed attractive results, especially for UC patients [11–14].

Numerous clinical trials have evidenced that FMT was characterized as a bacteria-driven therapy of maintaining remission (22%) of UC and preventing recurrence [15–17]. Additionally, Sun et al. [1] and Shi et al. [18] described that the donor selection, the administration type (e.g., enema, colonoscopy, gastroscopy, and nasogastric tube insertion), the time of FMT, and the microbiota relationship of recipients and donors lack systematic analysis. Given that there has been no systematic review focusing on UC subjects, we performed a systematic review with the most reliable evidence to assess the efficacy of FMT and establish a standard practical protocol of FMT administration for UC. Thus, we were supposed to take the Mayo score, SDI, Inflammatory Bowel Disease Questionnaire (IBDQ), and C-reactive protein (CRP) of pre- or post-FMT into account, and the FMT route of administration and donor selection were also considered for analysis.

## 2. Materials and Methods

### 2.1. Searching Strategies

We searched for articles published in PubMed and Web of Science with the following MeSH terms for fecal microbiota transplant: “fecal microbiota transplantation,” “faecal microbiota transplantation,” “fecal transfusion,” “fecal therapy,” “microbiota implant,” “flora implantation,” “bacteriotherapy,” and “FMT.” Then, the results were combined using “AND” with studies identified by alternatives for UC: “ulcerative colitis,” “UC,” “colitis,” “inflammatory bowel diseases,” and “IBD.” We enrolled all relevant articles to May 5, 2017, by reviewing the titles and abstracts about FMT in IBD or UC. Both parallel control and nonparallel control trials were included. Additionally, the reference lists of relevant articles were also scrutinized.

### 2.2. Data Collection and Quality Assessment of Studies

All study selections and data extractions were performed by two reviewers independently, and disagreements were resolved by discussion. The data contained authors, publication dates, countries, number of patients, severity and duration of UC, frequency of FMT, routes of FMT administrated, participants' characteristics, and remission rates, and response rates of follow-up time. Any discrepancies were resolved through further discussion. The studies had to meet the following inclusion criteria: (1) clinical trials, (2) randomized controlled trials (RCTs) and nonrandomized trials, and (3) patients of any age with UC who underwent FMT; the exclusion criteria are (1) duplicate publication, (2) animal or in vitro trials, (3) articles that included Crohn's disease (CD) patients, (4) language other than English, and (5) case reports, reviews, letters to the editor, and conference abstracts.

The Newcastle–Ottawa Scale (NOS) was adopted to assess the quality of included studies [19]. And eight items evaluated the quality of articles from different sides. In total, scores of ≥4 were considered as high qualities while scores of <4 were considered as poor qualities [20]. The total of eight answers generated the final scores for each study.

### 2.3. Data Synthesis and Statistical Analysis

The efficacy of FMT was assessed by clinical response and clinical remission. Clinical remission of UC was defined as Mayo score of <3; clinical response a decrease by 30% [13, 21]. And the evaluation index of the Mayo score baseline, SDI, IBDQ, and CRP were recorded. The subgroup analysis of FMT protocol in UC patients included FMT route of administration, donor selection, and fecal fresh or frozen status. Then, we analyzed them.

We assessed statistical heterogeneity with the value of *I*^2^. Values of *I*^2^ of 25, 50, and 75% were assumed to mean low, moderate, and high heterogeneity, respectively; values of *I*^2^ < 50% indicated low heterogeneity with a high quality of results. In the absence of statistical heterogeneity (*I*^2^ < 50%), we could use a fixed-effects model; otherwise, we used a random-effects model. Review Manager (version 5.2) and Stata (14.0) were applied for the analysis of the efficacy among RCTs and the subgroups, respectively. SPSS (version 24.0) and GraphPad Prism (version 6.0) were managed for statistical analysis and drawing. Pearson/Spearman's test was used for the correlation analysis. Results were expressed as mean ± SD or mean ± SEM. Statistical analysis was performed by variance (ANOVA) or Student's *t*-test [22, 23]. All tests were two-tailed, and a value of *P* < 0.05 was deemed statistically significant difference.

## 3. Results

### 3.1. Search Results and Quality Assessment

**Figure 1:**
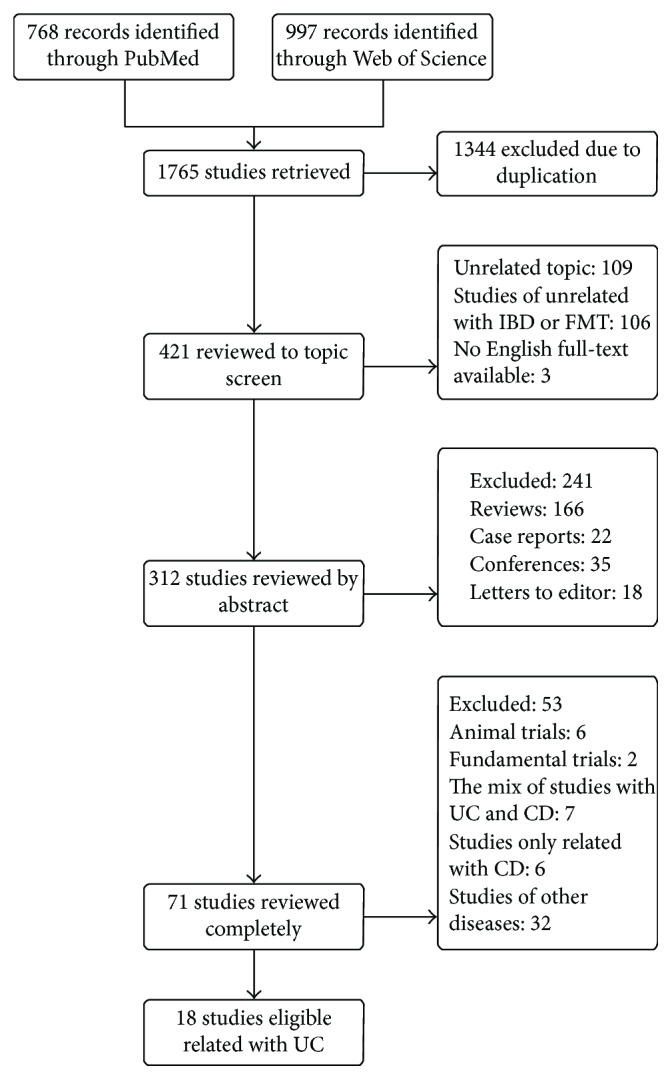
Flow diagram of included and excluded studies in this meta-analysis.

Our researches identified 421 articles with duplicate removal; of these, six trials focused only on CD [24–29], and eight case reports were related with UC patients [30–37]. A total of 25 studies related with UC patients. With the exception of seven studies that reported mixed patients (including UC and CD patients) [11–13, 38–41], all other eligible studies of 18 only included UC patients [42–58] (Figure 1). Among studies included, four trials belonged to randomized controlled trials (RCTs) [45, 51, 55, 57] and others were cohorts; 16 trials reported on the efficacy of FMT in UC patients. Ultimately, a total of 555 patients were enrolled, but only 446 patients met the inclusion criteria, of which 103 patients accepted the treatment of water, amoxicillin, fosfomycin, and metronidazole and 343 patients of FMT treatment. Studies enrolled were conducted in different countries: China, Netherland, Japan, America, Australia, Canada, Romania, Atlanta, and Chicago. And all eligible studies were considered as high qualities (each of >4 scores). All study demographic and clinical characteristics of UC patients are summarized in Table 1.

Among the 18 studies we included, the median score was 6.61 with a range of 4 to 8 for each item based on the NOS scoring system. All studies fell in “high-quality study” (those of ≥4 scores). Overall, the quality of included studies was deemed eligible. The qualities of each study included in our review are shown in Table 2.

### 3.2. The Efficacy of FMT and Placebo Treatment in UC

**Figure 2:**
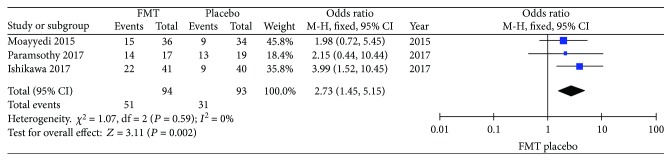
The efficacy of FMT and placebo treatment in UC.

A total of 16 articles were reported on the efficacy of FMT in UC patients, and the overall clinical response rate and clinical remission rate were 46.18 ± 25.08% and 28.96 ± 22.39%, respectively. Among four RCTs, only three trials included the FMT group and placebo group; besides, the study of Rossen et al. [55] divided all cases into FMT-autologous and FMT-donor fecal groups as control and experimental groups. No heterogeneity was found (*P* = 0.590, *I*^2^ = 0%), so a fixed-effect model was used; the FMT group had a possession of 94 among 187 cases, while 93 of 187 in the placebo group did not receive FMT treatment (e.g., water, amoxicillin, fosfomycin, and metronidazole). The patients that received FMT had a more apparent clinical response rate of 59.23% compared to the placebo group (39.13%) with a significantly statistical difference (OR: 2.73, 95% CI: 1.45–5.15, *P* = 0.002) (Figure 2).

### 3.3. The Relation of Evaluation Index and Efficacy at Pre-/Post-FMT in UC

The evaluation index of FMT efficacy in our review included the Mayo score, SDI, IBDQ, and CRP. We analyzed their changes at pre-FMT and post-FMT; meanwhile, we demonstrated that their baseline impacted the clinical response or remission rate at the intervention of fecal transplantation.

**Figure 3:**
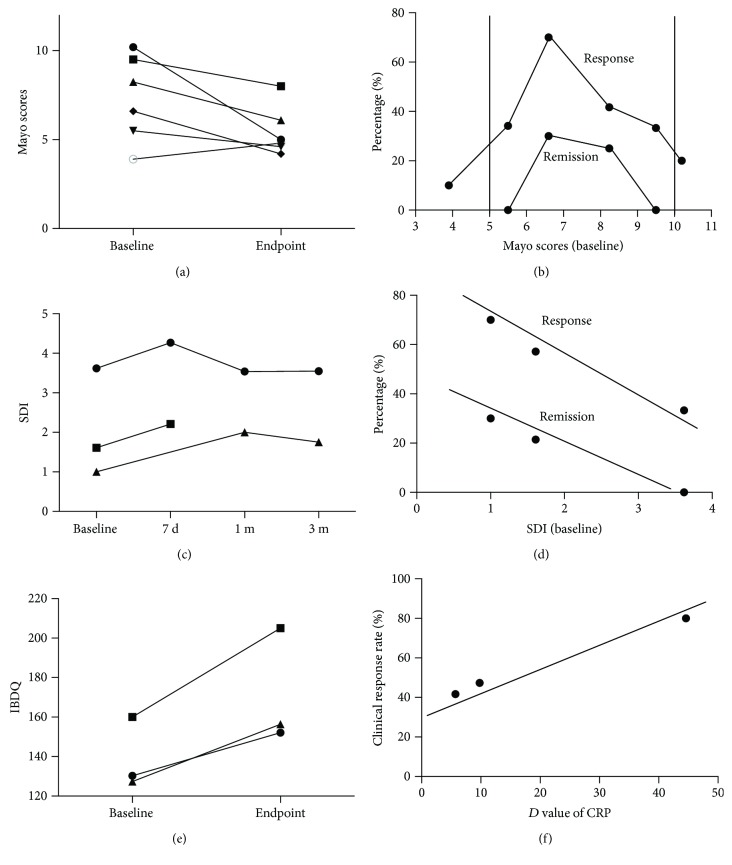
The relation of the evaluation index and efficacy at pre-/post-FMT in UC. (a) Mayo scores decreased to 5 points at the endpoint of FMT among almost all of the studies. (b) The optimal range of the Mayo score baseline was 6–9 at the treatment of FMT. (c) The SDI of post-FMT reached up to the highest level at seven days to one month. (d) The SDI baseline had significant negative correlations with clinical response and remission of FMT in UC patients (*R*^response^ = −0.992, *P*^response^ = 0.08; *R*^remission^ = −0.998, *P*^remission^ = 0.036). (e) IBDQ had an increased tendency after FMT. (f) The decrease in CRP (*D* value of CRP, mg/L) has a significant positive correlation with the clinical response rate after FMT (*R*^response^ = 0.99, *P* = 0.027).

Six of eighteen studies reported the Mayo score of UC patients [21, 42, 50–52, 57] and included 108 cases. A total Mayo score of <3 was defined as clinical remission of UC, and a decrease of >30% was the clinical response of UC; Mayo scores equal to 3, 5, and 10 were assumed to represent mild, moderate, and severe active UC.  Figure 3(a) shows that the Mayo score decreased to 5 at the endpoint of FMT among almost all of the studies, which were characterized as mild–moderate activity of UC. Interestingly, the Mayo score in the combination of the FMT and pectin groups (Wei et al. [57]) decreased to 2.25 ± 0.75 at the endpoint with clinical remission; at the same time, the FMT group showed a lower clinical response rate of 34.86% and a remission rate of 13.75% compared to the FMT + pectin group with a response rate of 70% and a remission rate of 30%.  Figure 3(b) shows that the optimal range of the Mayo score baseline was 6–9 in terms of the clinical response or clinical remission of FMT.

There were three articles reported on the Shannon diversity index (SDI) with a measure of the colonic bacterial diversity on pre-FMT and post-FMT in UC patients [21, 43, 57]. Only 20 patients were enrolled in this analysis. The SDI of post-FMT reached the highest level from seven days to one month (Figure 3(c)). In terms of clinical response and remission, both of them had significant negative correlations with an SDI baseline of pre-FMT in UC patients (Pearson: *R*^response^ = −0.992, *P*^response^ = 0.08; *R*^remission^ = −0.998, *P*^remission^ = 0.036) (Figure 3(d)).

Only three trials were related to IBDQ with a total of 94 cases [51, 55, 57]. It showed an increased tend at endpoint of IBDQ (Figure 3(e)). And four studies reported on CRP value, of which three trials were eligible [49, 51, 58]. The decrease of CRP (*D*-value of CRP, mg/L) has a significant positive correlation with clinical response rate after FMT (Pearson: *R*^response^ = 0.99, *P* = 0.027) (Figure 3(f)).

### 3.4. Subgroup Analysis of FMT Optimal Administration

**Figure 4:**
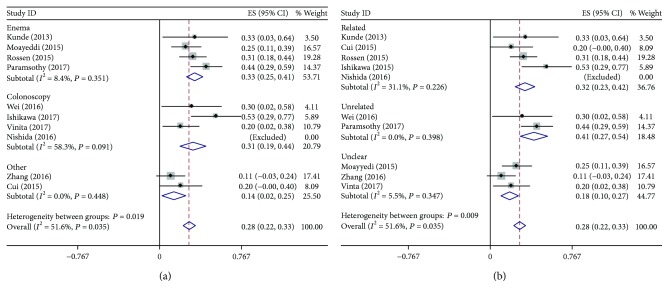
Subgroup analysis of FMT optimal administration: (a) the difference between enema and colonoscopy groups; (b) the difference between related and unrelated donor groups.

Ten articles (each ≥ 10 cases) were obtained and reviewed for potential eligibility in our subgroup analysis based on the clinical remission rate (the clinical response rate had a significant heterogeneity). Our first subgroup analysis compared the efficacy of the FMT route of enema and colonoscopy. Four trials (*n* = 135) [47, 51, 54, 55] reported on enema as a manner of FMT, four trials (*n* = 88) on colonoscopy [45, 46, 52, 57], and two on other manners of nasogastric tube insertion, nasojejunal tube insertion, gastroscopy, and esophagogastroduodenoscopy [43, 58]. As shown in Figure 4(a), there was low or moderate heterogeneity in each group (enema: *P* = 0.35, *I*^2^ = 8.4%; colonoscopy: *P* = 0.09, *I*^2^ = 58.3%). The rate of clinical remission in the enema group was 33.37% (95% CI: 0.25–0.41), and that in the colonoscopy group was 25.74% (95% CI: 0.19–0.44) (Table 3). The clinical efficacy of the colonoscopy route of administration was similar to that of enema.

Our second subgroup analysis compared the efficacy of related fecal donor and unrelated donor applied in the FMT of UC patients. Five articles based on related donor [43, 45, 47, 52, 55] and two based on unrelated donor [54, 57] were available and enrolled. A total of 131 subjects in the related donor group had a clinical remission of 27.79% with a low homogeneity (*P* = 0.23, *I*^2^ = 31.1%), and the unrelated donor group (*n* = 51) had a higher clinical remission of 36.95% with a low homogeneity (*P* = 0.4, *I*^2^ = 0.0%). All data are showed in Figure 4(b) and Table 3.

Finally, data on the bacterial fluid status were represented based on fresh fecal and frozen fecal transplantation. UC patients among six studies (*n* = 133) [45, 48, 51, 52, 57, 58] used fresh feces with an overall clinical remission rate of 25.30%. As for the frozen fecal group, only Paramsothy et al. [54] claimed a remission rate of 43.9% (Table 3).

## 4. Discussion

To date, this article comprehensively summarized the efficacy and evaluation index in the treatment of FMT among UC patients, which identified 16 articles showing the clinical efficacy of FMT for our analysis. The use of FMT for the management of UC patients resulted in a higher efficacy of 59.23% compared to the meta-analysis of Costello et al. (49%) [59]. Importantly, the FMT route of colonoscopy administration (25.74%) is consistent with the study of Sun et al [1]. showing colonoscopy administration of 29.8%, and the unrelated donor (36.95%) had a significant effect for UC patients. We also mainly discussed that the baseline of the Mayo score and SDI played favorable roles on UC patients, influencing the clinical response and remission of FMT administration. Nonetheless, the results from pre-/post-FMT and subgroup analysis still remained controversial.

### 4.1. Evaluation Index

The Mayo score was considered as the comprehensive system in almost all aspects, including defecation frequency, hematochezia, endoscopic evaluation, and physicians' score [60]. A score of <3 was considered as a clinical remission, and a reduction of >30% from baseline was a clinical response [57, 61, 62]. Our analysis showed that the Mayo score significantly decreased to 5 points at the endpoint after FMT, which was consistent with the mild–moderate activity (score of 3–10) of UC (Figure 3(a)); however, Wei et al. [57] investigated the effects of the combination therapy of FMT and pectin with a lower Mayo score and a higher efficacy, which explains why pectin delayed the loss of diversity of transplanted gut flora enhancing the effects of FMT in UC cases. Therefore, FMT needs to use adjunctive therapies to improve its effect. Moreover, the Mayo score baseline of 6–9 holds a significant clinical response. Both of the above results explained the optimal requirement of FMT in UC patients from the Mayo score point of view, which were linked to the complexity of pathogenesis in UC patients.

Moreover, our review has evidenced that, on the one hand, FMT had a short-time effect for the regulation of intestinal flora diversity at the following 7 days at the treatment of FMT, but one month later, bacterial diversity began to fall. Notably, the reason for the transient effect of FMT treatment is still bewildering. Furusawa et al. [63] explained that the following factors might influence the result in detail: the time of FMT administration [57], bowel preparation, and antibiotic usage [64, 65]. Nevertheless, the higher the baseline of the Shannon index, the higher the clinical response or remission rate (Figure 3(d)). The verdict of the SDI baseline will provide the proof of assessment of the efficacy for the physician. On the other hand, the SDI of post-FMT reached up to the highest level between 7 days and one month, which was considered as the early efficacy of FMT assessment. The Toronto Consensus of 2015 [66] has evidenced that the time-points of early efficacy of 5-amino salicylic acid, corticosteroids, and anti-TNF mAbs were 1–2 months, 0.5 month, and 2–3 months, separately. Thus, it can be seen that FMT administration is superior to traditional therapies in the treatment of UC due to the shorter assessment time of efficacy.

### 4.2. Subgroup Analysis

In our review, we found that colonoscopy administration appeared to have a superiority in treatment of UC via FMT administration. Firstly, considering the complex pathogenesis and clinical characteristics of UC, it was likely to start in the rectum and extend to the proximal colon; it therefore was best targeted by lower gastrointestinal delivery of FMT compared to upper gastrointestinal delivery (e.g., nasogastric tube insertion, nasojejunal tube insertion, gastroscopy, and esophagogastroduodenoscopy) [1]. Secondly, our results showed that it was as effective as enema administration (25.74% versus 33.37%). And Ishikawa et al. [45] reported results that the administration of colonoscopy had a clinical remission rate of 52.94% in UC patients, and Paramsothy et al. [54] posited that the rate of clinical remission peaked at 43.9% via enema. At the same time, most patients in our enrolled articles often received unclear preintervention impacting the effect of different routes of FMT administration, and we had a difficulty in obtaining complete data and precise analysis after FMT via colonoscopy or enema. Moreover, some studies had suggested a slight superiority of FMT in CDI patients via colonoscopy but without sufficient evidence [9]. Most importantly, colonoscopy could not only deliver enough volume suspensions directly into the site of inflammation in the colon but also visualize the relevant gut lesion achieving two things at one stroke; in addition, there was intolerance to retention enema in some studies compared to colonoscopy due to multiple times of administration and the patients' tolerance itself. Therefore, the FMT treatment via colonoscopy will have a very good prospect in UC patients. However, additional high-quality and better-designed researches are needed for further investigating this procedural aspect.

In the review of fecal donor selection, it still remained debatable in FMT application. The phase II trial of Rossen et al. [55] had already reported that there was no statistically significant difference in clinical and endoscopic remission between UC patients who received FMT from unrelated and related donors. And Costello et al. [67] showed that the clinical remission of UC patients receiving an unrelated fecal donor of 50% was higher than for a related fecal donor of 17%. It was similar to our results of unrelated donor having a mildly higher clinical remission rate of 36.95%. As is generally known, to a great extent, related donors share common microbial species with the recipients (UC patients), which minimized the risk of adverse events associated with FMT administration. Nevertheless, the recipients who received related feces suffered from a recurrence of UC in patients; no data regarding recipients receiving unrelated feces exists in this issue. Then, unrelated donors had advantages of lower costs and simpler process compared to related donors. In light of these observations, unrelated feces may thus be preferable for UC patients.

### 4.3. Limitation

FMT has concerned some ethical and medical technology standardization issues largely other than that seen as an upcoming treatment strategy for UC patients. Firstly, some patients were resistant to the use of FMT as a viable treatment option, unless they were only under the circumstance of no other way [68]. Moreover, a matter that should not be neglected was the essence of FMT treatment, organ transplantation, or biological therapy. Finally, a standard and detailed FMT procedure in clinical trials for researchers is still absent.

Our analysis involved several limitations in this review: Firstly, our statistical analysis was based on individual articles because we did not have detailed data of each case for all the trials; secondly, the small number of cases among some trials was included by our review, and the evidence might be weak. Thirdly, among the articles about the evaluation index (e.g., Mayo score, SDI, IBDQ, and CRP), less than half of the total studies were enrolled. Finally, several included studies received the drug therapy before or after FMT and may influence the accuracy and specify our results.

## 5. Conclusion

We have demonstrated that FMT was an alternative therapy for UC with a certain efficacy. Then, the combination therapy of FMT and a lower baseline of microbial richness probably further contribute to the curative effect in preclinical and clinical practices.

## Figures and Tables

**Table 1:** Demographic and clinical features at enrollment in clinical trials.

Source	Year	Country	RCT	No. of enrolled patients (missing)^∗^	Age	Severity of UC	Duration time of UC (years)	Concomitant drug treatment^∗^	FMT delivery	FMT frequency	Follow-up (months)	Assessment
Angelberger	2013	Austria	No	5	34.2 ± 12.56	Moderately, severely	4.1 ± 3.37	Yes	Nasojejunal tube and enema	Single	3	Mayo scores, CRP, fecal calprotectin
Kahn	2013	Chicago	No	44 (51)	39	Mildly, moderately, severely	7	NA	Colonoscopy (77%, enema (20%), nasogastric tube (3%)	Single	1	NA
Kunde	2013	Atlanta	No	10	15.2 ± 4.44	Mildly, moderately	3.51 ± 2.63	Yes	Enema	Single	2	PUCAI
Patrizia	2013	Austria	No	6	36.5 ± 12.1	NA	5.5 ± 4.32	Yes	Colonoscopy	Single	3	Fecal calprotectin, Mayo scores, CRP
Cui	2015	China	No	15	31.67 ± 10.22	Moderately, severely	4.2 ± 2.91	Yes	Colonoscopy	1 single, 5 twice	3	CRP, ESR
Damman	2015	America	No	7	41.14 ± 15.5	Mildly, moderately	16.57 ± 13.14	Yes	Colonoscopy	Single	NA	DSI, fecal calprotectin, UCDAI
Moayyedi	2015	Canada	Yes	FMT: 36 (2), placebo: 34 (3)	FMT: 42.2 ± 15.0, placebo: 35.8 ± 12.1,	NA	FMT: 7.9 ± 5.6, placebo: 7.0 ± 6.8,	Yes	Enema	6 times	12	Mayo scores, IBDQ, EQ-5D, CRP, ESR
Rossen	2015	Netherlands	Yes	FMT-D: 23, FMT-A: 25	NA	Mildly, moderately	NA	No	Enema	>Single	12	Mayo scores, SCCAI, IBDQ
Suskind	2015	America	No	4	14.5 ± 1.73	Mildly, moderately	1	Yes	Nasogastric tube	Single	3	PUCAI, CRP, stool calprotectin
Laszlo	2016	Romania	No	5	NA	Moderately, severely	3 ± 1	Yes	Colonoscopy	NA	10	NA
Nishida	2016	Japan	No	41 (16)	39.6 ± 16.9	Mildly, moderately	7.6 ± 8.6	Yes	Colonoscopy	Single	2	Mayo scores, CRP
Oprita	2016	Romania	No	5 (28)	NA	Moderately: 1, severely: 4	NA	Yes	Nasojejunal tube (40%), colonoscopy (60%)	Single 1, twice 3	3	NA
Zhang	2016	China	No	19	39.2 ± 14.1	Moderately: 8, severely: 11	8.0 ± 5.8	4 yes, 15 no	Nasogastric tube	Single	3	CRP, cytokines
Wei	2016	China	Yes	FMT: 10, FMT + pectin: 10	FMT: 43.50 ± 15, FMT + pectin: 37.40 ± 9.92	NA	NA	Yes	Colonoscopy	NA	3	Mayo score, ESR, CRP, IBDQ, fecal calprotectin, Shannon index
Ishikawa	2017	Japan	Yes	FMT: 17 (4), AFM: 19 (1)	FMT: 40.4 ± 14.2, AFM: 44.7 ± 14.9	Mildly, severely	FMT: 7.8 ± 8.4, AFM: 7.0 ± 8.0	Yes	Colonoscopy	Single	1	CAI, Mayo scores
Mizuno	2017	Japan	No	10	NA	Moderately: 5, severely: 5	4.5	Yes	Colonoscopy	Single	3	Mayo scores
Vinita	2017	America	No	20	38.4 ± 12.6	NA	NA	Yes	Colonoscopy	Single	3	Mayo scores
Paramsothy	2017	Australia	Yes	FMT: 41(1), placebo: 40(3)	35.6	NA	5.8	Yes	Enema	NA	2	Mayo scores, IBDQ

Note: RCT: randomized controlled trials; NA: not available (NA); AFM: amoxicillin, fosfomycin, and metronidazole; HBI: Harvey–Bradshaw Index; MRI: magnetic resonance imaging; CT: computed tomography scan (CT); ESR: erythrocyte sedimentation rate; CRP: C-reactive protein; PCDAI: pediatric Crohn's disease activity index; CBC: complete blood count; BCI: Bray–Curtis index; sIBDQ: short inflammatory bowel disease questionnaire; CDEIS: Crohn's Disease Endoscopic Index of Severity; QoL: quality of life; CDAI: Crohn's disease activity index; UCCS: Ulcerative Colitis Clinical Score; SES-CD: simplified endoscopic activity score; PUCAI: pediatric UC activity index; EQ-5D: EuroQol; SCCAI: simple clinical colitis activity index; IBDQ inflammatory bowel disease questionnaire. ^∗^Concomitant drug treatment: infliximab, cyclosporine, thiopurine, methotrexate, steroid, and so on.

**Table 2:** Quality assessment of studies enrolled in UC.

Author	Year	Representativeness of the exposed cohort	Selection of the nonexposed cohort	Ascertainment of exposure	No demonstration of interesting outcome at start of study	Control for important factor or additional factor	Assessment of outcome	Enough follow-up of outcome	Adequacy of follow up of cohorts	Total quality scores
Angelberger	2013	1	0	1	1	1	1	1	0	6
Kahn	2013	1	0	1	1	1	0	0	0	4
Kunde	2013	1	0	1	1	2	1	0	0	6
Patrizia	2013	1	0	1	1	1	1	1	1	7
Cui	2015	1	0	1	1	2	1	1	1	8
Damman	2015	1	0	1	1	1	1	0	0	5
Moayyedi	2015	1	1	1	1	0	1	1	1	7
Rossen	2015	1	1	1	1	1	1	1	1	8
Suskind	2015	1	0	1	1	1	1	0	0	5
Laszlo	2016	1	0	1	1	1	1	1	1	7
Nishida	2016	1	0	1	1	2	1	0	1	7
Oprita	2016	1	0	1	1	1	1	1	0	6
Zhang	2016	1	0	1	1	1	1	1	1	7
Wei	2016	1	1	1	1	1	1	1	1	8
Ishikawa	2017	1	1	1	1	2	1	0	0	7
Mizuno	2017	1	0	1	1	1	1	1	1	7
Vinita	2017	1	0	1	1	0	1	1	1	6
Paramsothy	2017	1	1	1	1	1	1	1	1	8

Total score, 9; ≤4, poor quality; >4, good quality.

**Table 3:** Subgroup analysis for FMT in UC patients (cases > 10).

Subgroups	Number of studies (*n*)	No. of enrolled patients	Remission rate (*M* ± SEM, %)	The value of *P*^∗∗^	95% confidence interval	Tests of homogeneity	
						*P*	*I* ^2^
Route of administration							
Enema	4	135	33.37 ± 3.93	*P* > 0.05	0.25–0.41	0.35	8.4%
Colonoscopy	4	88	25.74 ± 11.00		0.19–0.44	0.09	58.3%
Other^∗^	2	34	15.98 ± 5.45		0.02–0.25	0.45	0.0%
Donor relationship							
Related	5	131	27.79 ± 8.63	*P* > 0.05	0.23–0.42	0.23	31.1%
Unrelated	2	51	36.95 ± 6.95		0.27–0.54	0.40	0.0%
Unclear	3	75	18.51 ± 4.24		0.10–0.27	0.35	5.5%
Bacterial fluid status							
Fresh	6	133	25.30 ± 7.55	*P* > 0.05	NA	NA	NA
Frozen	1	41	43.9		NA	NA	NA
Unclear	3	83	24.23 ± 3.5		NA	NA	NA

Note: 95% CI: 95% confidence interval; NA: not available. ^∗^Others: nasogastric tube insertion, gastroscopy, nasojejunal tube insertion, esophagogastroduodenoscopy; ^∗∗^*P* means pairwise comparison of subgroup.

## References

[1] Sun D., Li W., Li S. (2016). Fecal microbiota transplantation as a novel therapy for ulcerative colitis: a systematic review and meta-analysis. *Medicine*.

[2] Danese S., Fiocchi C. (2011). Ulcerative colitis. *The New England Journal of Medicine*.

[3] Stange E. F., Travis S. P., Vermeire S. (2008). European evidence-based consensus on the diagnosis and management of ulcerative colitis: definitions and diagnosis. *Journal of Crohn's and Colitis*.

[4] Burisch J., Munkholm P. (2013). Inflammatory bowel disease epidemiology. *Current Opinion in Gastroenterology*.

[5] Cammarota G., Ianiro G., Cianci R., Bibbò S., Gasbarrini A., Currò D. (2015). The involvement of gut microbiota in inflammatory bowel disease pathogenesis: potential for therapy. *Pharmacology & Therapeutics*.

[6] Macfarlane S., Macfarlane G. T. (2004). Bacterial diversity in the human gut. *Advances in Applied Microbiology*.

[7] Eiseman B., Silen W., Bascom G. S., Kauvar A. J. (1958). Fecal enema as an adjunct in the treatment of pseudomembranous enterocolitis. *Surgery*.

[8] Broecker F., Kube M., Klumpp J. (2013). Analysis of the intestinal microbiome of a recovered *Clostridium difficile* patient after fecal transplantation. *Digestion*.

[9] Kassam Z., Lee C. H., Yuan Y., Hunt R. H. (2013). Fecal microbiota transplantation for *clostridium difficile* infection: systematic review and meta-analysis. *The American Journal of Gastroenterology*.

[10] Cammarota G., Ianiro G., Gasbarrini A. (2014). Fecal microbiota transplantation for the treatment of clostridium difficile infection: a systematic review. *Journal of Clinical Gastroenterology*.

[11] Khoruts A., Rank K. M., Newman K. M. (2016). Inflammatory bowel disease affects the outcome of fecal microbiota transplantation for recurrent clostridium difficile infection. *Clinical Gastroenterology and Hepatology*.

[12] Fischer M., Kao D., Kelly C. (2016). Fecal microbiota transplantation is safe and efficacious for recurrent or refractory *clostridium difficile* infection in patients with inflammatory bowel disease. *Inflammatory Bowel Diseases*.

[13] Wei Y., Zhu W., Gong J. (2015). Fecal microbiota transplantation improves the quality of life in patients with inflammatory bowel disease. *Gastroenterology Research and Practice*.

[14] Mattner J., Schmidt F., Siegmund B. (2016). Faecal microbiota transplantation-a clinical view. *International Journal of Medical Microbiology*.

[15] Sood A., Midha V., Makharia G. K. (2009). The probiotic preparation, VSL#3 induces remission in patients with mild-to-moderately active ulcerative colitis. *Clinical Gastroenterology and Hepatology*.

[16] Tursi A., Brandimarte G., Papa A. (2010). Treatment of relapsing mild-to-moderate ulcerative colitis with the probiotic VSL#3 as adjunctive to a standard pharmaceutical treatment: a double-blind, randomized, placebo-controlled study. *The American Journal of Gastroenterology*.

[17] Colman R. J., Rubin D. T. (2014). Fecal microbiota transplantation as therapy for inflammatory bowel disease: a systematic review and meta-analysis. *Journal of Crohn's and Colitis*.

[18] Shi Y., Dong Y., Huang W., Zhu D., Mao H., Su P. (2016). Fecal microbiota transplantation for ulcerative colitis: a systematic review and meta-analysis. *PLoS One*.

[19] Gong Q., Janowski M., Luo M. (2017). Efficacy and adverse effects of atropine in childhood myopia: a meta-analysis. *JAMA Ophthalmology*.

[20] Cao Y., Ding Z., Han C., Shi H., Cui L., Lin R. (2017). Efficacy of mesenchymal stromal cells for fistula treatment of Crohn’s disease: a systematic review and meta-analysis. *Digestive Diseases and Sciences*.

[21] Kump P. K., Gröchenig H. P., Lackner S. (2013). Alteration of intestinal dysbiosis by fecal microbiota transplantation does not induce remission in patients with chronic active ulcerative colitis. *Inflammatory Bowel Diseases*.

[22] Lin R., Ding Z., Ma H. (2015). In vitro conditioned bone marrow-derived mesenchymal stem cells promote de novo functional enteric nerve regeneration, but not through direct-transdifferentiation. *Stem Cells*.

[23] Lin R., Murtazina R., Cha B. (2011). D-glucose acts via sodium/glucose cotransporter 1 to increase NHE3 in mouse jejunal brush border by a Na+/H+ exchange regulatory factor 2-dependent process. *Gastroenterology*.

[24] Xu L., Zhang T., Cui B. (2016). Clinical efficacy maintains patients’ positive attitudes toward fecal microbiota transplantation. *Medicine*.

[25] Suskind D. L., Brittnacher M. J., Wahbeh G. (2015). Fecal microbial transplant effect on clinical outcomes and fecal microbiome in active Crohn’s disease. *Inflammatory Bowel Diseases*.

[26] Cui B., Feng Q., Wang H. (2015). Fecal microbiota transplantation through mid-gut for refractory Crohn’s disease: safety, feasibility, and efficacy trial results. *Journal of Gastroenterology and Hepatology*.

[27] Brittnacher M. J., Heltshe S. L., Hayden H. S. (2016). GUTSS: an alignment-free sequence comparison method for use in human intestinal microbiome and fecal microbiota transplantation analysis. *PLoS One*.

[28] Vaughn B. P., Vatanen T., Allegretti J. R. (2016). Increased intestinal microbial diversity following fecal microbiota transplant for active Crohn’s disease. *Inflammatory Bowel Diseases*.

[29] Vermeire S., Joossens M., Verbeke K. (2012). Pilot study on the safety and efficacy of faecal microbiota transplantation in refractory Crohn’s disease. *Gastroenterology*.

[30] Seth A. K., Rawal P., Bagga R., Jain P. (2016). Successful colonoscopic fecal microbiota transplantation for active ulcerative colitis: first report from India. *Indian Journal of Gastroenterology*.

[31] Shimizu H., Arai K., Abe J. (2016). Repeated fecal microbiota transplantation in a child with ulcerative colitis. *Pediatrics International*.

[32] Brace C., Gloor G. B., Ropeleski M., Allen-Vercoe E., Petrof E. O. (2014). Microbial composition analysis of *Clostridium difficile* infections in an ulcerative colitis patient treated with multiple fecal microbiota transplantations. *Journal of Crohn's and Colitis*.

[33] Aratari A., Cammarota G., Papi C. (2015). Fecal microbiota transplantation for recurrent C. difficile infection in a patient with chronic refractory ulcerative colitis. *Journal of Crohn's and Colitis*.

[34] Kao D., Madsen K. (2013). Fecal microbiota transplantation (FMT) in the treatment of inflammatory bowel disease (IBD): a case report. *American Journal of Gastroenterology*.

[35] Vandenplas Y., Veereman G., van der Werff ten Bosch J. (2015). Fecal microbial transplantation in early-onset colitis: caution advised. *Journal of Pediatric Gastroenterology and Nutrition*.

[36] Kumagai H., Yokoyama K., Imagawa T. (2016). Failure of fecal microbiota transplantation in a three-year-old child with severe refractory ulcerative colitis. *Pediatric Gastroenterology, Hepatology & Nutrition*.

[37] Ni X., Fan S., Zhang Y. (2016). Coordinated hospital-home fecal microbiota transplantation via percutaneous endoscopic cecostomy for recurrent steroid-dependent ulcerative colitis. *Gut and Liver*.

[38] Vermeire S., Joossens M., Verbeke K. (2016). Donor species richness determines Faecal microbiota transplantation success in inflammatory bowel disease. *Journal of Crohn's and Colitis*.

[39] Chin S. M., Sauk J., Mahabamunuge J., Kaplan J. L., Hohmann E. L., Khalili H. (2017). Fecal microbiota transplantation for recurrent *Clostridium difficile* infection in patients with inflammatory bowel disease: a single-center experience. *Clinical Gastroenterology and Hepatology*.

[40] Hourigan S. K., Chen L. A., Grigoryan Z. (2015). Microbiome changes associated with sustained eradication of *Clostridium difficile* after single faecal microbiota transplantation in children with and without inflammatory bowel disease. *Alimentary Pharmacology & Therapeutics*.

[41] Fischer M., Kao D., Mehta S. R. (2016). Predictors of early failure after fecal microbiota transplantation for the therapy of *clostridium difficile* infection: a multicenter study. *The American Journal of Gastroenterology*.

[42] Angelberger S., Reinisch W., Makristathis A. (2013). Temporal bacterial community dynamics vary among ulcerative colitis patients after fecal microbiota transplantation. *The American Journal of Gastroenterology*.

[43] Cui B., Li P., Xu L. (2015). Step-up fecal microbiota transplantation strategy: a pilot study for steroid-dependent ulcerative colitis. *Journal of Translational Medicine*.

[44] Damman C. J., Brittnacher M. J., Westerhoff M. (2015). Low level engraftment and improvement following a single colonoscopic administration of fecal microbiota to patients with ulcerative colitis. *PLoS One*.

[45] Ishikawa D., Sasaki T., Osada T. (2017). Changes in intestinal microbiota following combination therapy with fecal microbial transplantation and antibiotics for ulcerative colitis. *Inflammatory Bowel Diseases*.

[46] Jacob V., Crawford C., Cohen-Mekelburg S. (2017). Single delivery of high-diversity fecal microbiota preparation by colonoscopy is safe and effective in increasing microbial diversity in active ulcerative colitis. *Inflammatory Bowel Diseases*.

[47] Kahn S. A., Vachon A., Rodriquez D. (2013). Patient perceptions of fecal microbiota transplantation for ulcerative colitis. *Inflammatory Bowel Diseases*.

[48] Kunde S., Pham A., Bonczyk S. (2013). Safety, tolerability, and clinical response after fecal transplantation in children and young adults with ulcerative colitis. *Journal of Pediatric Gastroenterology and Nutrition*.

[49] Laszlo M., Ciobanu L., Andreica V., Pascu O. (2016). Fecal transplantation indications in ulcerative colitis. Preliminary study. *Clujul Medical*.

[50] Mizuno S., Nanki K., Matsuoka K. (2017). Single fecal microbiota transplantation failed to change intestinal microbiota and had limited effectiveness against ulcerative colitis in Japanese patients. *Intestinal Research*.

[51] Moayyedi P., Surette M. G., Kim P. T. (2015). Fecal microbiota transplantation induces remission in patients with active ulcerative colitis in a randomized controlled trial. *Gastroenterology*.

[52] Nishida A., Imaeda H., Ohno M. (2017). Efficacy and safety of single fecal microbiota transplantation for Japanese patients with mild to moderately active ulcerative colitis. *Journal of Gastroenterology*.

[53] Oprita R., Bratu M., Oprita B., Diaconescu B. (2016). Fecal transplantation – the new, inexpensive, safe, and rapidly effective approach in the treatment of gastrointestinal tract diseases. *Journal of Medicine and Life*.

[54] Paramsothy S., Kamm M. A., Kaakoush N. O. (2017). Multidonor intensive faecal microbiota transplantation for active ulcerative colitis: a randomised placebo-controlled trial. *The Lancet*.

[55] Rossen N. G., Fuentes S., van der Spek M. J. (2015). Findings from a randomized controlled trial of fecal transplantation for patients with ulcerative colitis. *Gastroenterology*.

[56] Suskind D. L., Singh N., Nielson H., Wahbeh G. (2015). Fecal microbial transplant via nasogastric tube for active pediatric ulcerative colitis. *Journal of Pediatric Gastroenterology and Nutrition*.

[57] Wei Y., Gong J., Zhu W. (2016). Pectin enhances the effect of fecal microbiota transplantation in ulcerative colitis by delaying the loss of diversity of gut flora. *BMC Microbiology*.

[58] Zhang T., Cui B., Li P. (2016). Short-term surveillance of cytokines and C-reactive protein cannot predict efficacy of fecal microbiota transplantation for ulcerative colitis. *PloS One*.

[59] Costello S. P., Soo W., Bryant R. V., Jairath V., Hart A. L., Andrews J. M. (2017). Systematic review with meta-analysis: faecal microbiota transplantation for the induction of remission for active ulcerative colitis. *Alimentary Pharmacology & Therapeutics*.

[60] Nakazato Y., Naganuma M., Sugimoto S. (2017). Endocytoscopy can be used to assess histological healing in ulcerative colitis. *Endoscopy*.

[61] Brandt L. J., Aroniadis O. C., Mellow M. (2012). Long-term follow-up of colonoscopic fecal microbiota transplant for recurrent *Clostridium difficile* infection. *The American Journal of Gastroenterology*.

[62] Kornbluth A., Sachar D. B., The Practice Parameters Committee of the American College of Gastroenterology (2010). Ulcerative colitis practice guidelines in adults: American College Of Gastroenterology, Practice Parameters Committee. *The American Journal of Gastroenterology*.

[63] Furusawa Y., Obata Y., Fukuda S. (2013). Commensal microbe-derived butyrate induces the differentiation of colonic regulatory T cells. *Nature*.

[64] D'Haens G., Sandborn W. J., Feagan B. G. (2007). A review of activity indices and efficacy end points for clinical trials of medical therapy in adults with ulcerative colitis. *Gastroenterology*.

[65] Irvine E. J., Feagan B., Rochon J. (1994). Quality of life: a valid and reliable measure of therapeutic efficacy in the treatment of inflammatory bowel disease. *Gastroenterology*.

[66] Bressler B., Marshall J. K., Bernstein C. N. (2015). Clinical practice guidelines for the medical management of non-hospitalized ulcerative colitis: the Toronto Consensus. *Gastroenterology*.

[67] Costello S., Waters O., Bryant R. (2017). OP036 Short duration, low intensity pooled faecal microbiota transplantation induces remission in patients with mild-moderately active ulcerative colitis: a randomised controlled trial. *Journal of Crohn’s and Colitis*.

[68] Glauser W. (2011). Risk and rewards of fecal transplants. *CMAJ*.

